# Generalized Fragment Picking in Rosetta: Design, Protocols and Applications

**DOI:** 10.1371/journal.pone.0023294

**Published:** 2011-08-24

**Authors:** Dominik Gront, Daniel W. Kulp, Robert M. Vernon, Charlie E. M. Strauss, David Baker

**Affiliations:** 1 Faculty of Chemistry, University of Warsaw, Warsaw, Poland; 2 Los Alamos National Laboratory, Bioscience Division, Los Alamos, New Mexico, United States of America; 3 Program in Molecular Structure and Function, Hospital For Sick Children, Toronto, Canada; 4 Department of Biochemistry, Howard Hughes Medical Institute, University of Washington, Seattle, Washington, United States of America; University of South Florida College of Medicine, United States of America

## Abstract

The Rosetta de novo structure prediction and loop modeling protocols begin with coarse grained Monte Carlo searches in which the moves are based on short fragments extracted from a database of known structures. Here we describe a new object oriented program for picking fragments that greatly extends the functionality of the previous program (nnmake) and opens the door for new approaches to structure modeling. We provide a detailed description of the code design and architecture, highlighting its modularity, and new features such as extensibility, total control over the fragment picking workflow and scoring system customization. We demonstrate that the program provides at least as good building blocks for *ab-initio* structure prediction as the previous program, and provide examples of the wide range of applications that are now accessible.

## Introduction

**Figure 1:**
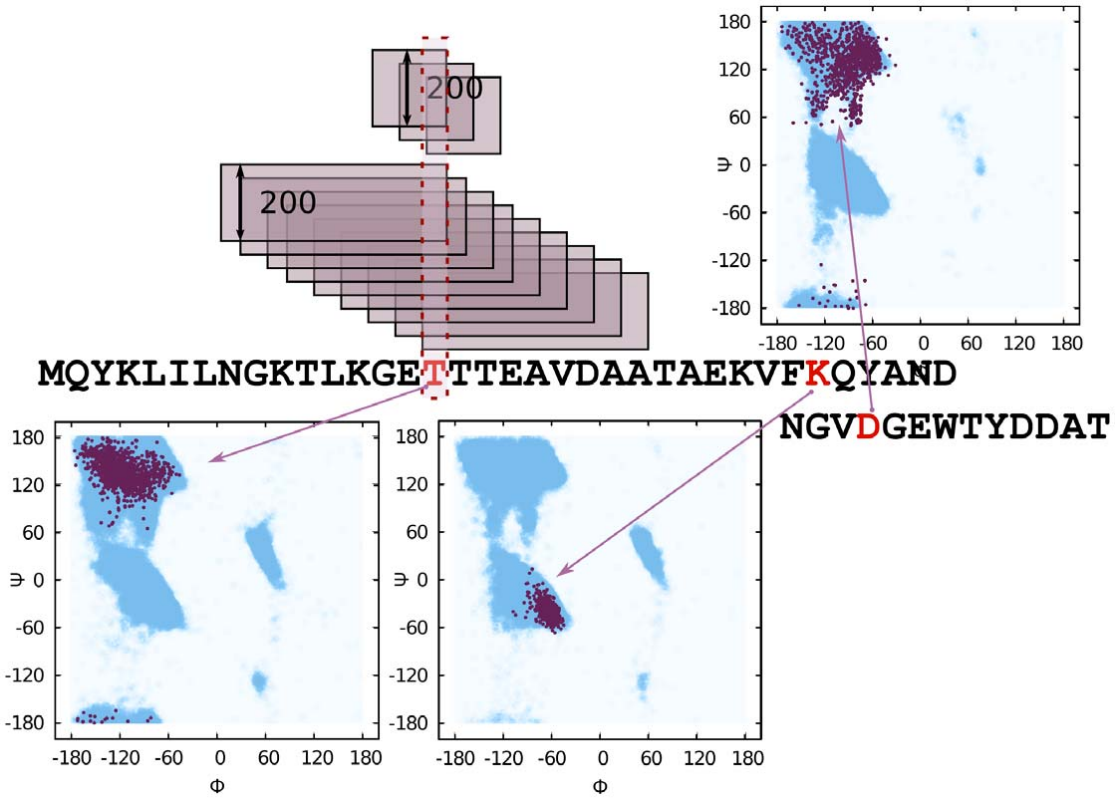
Overlapping fragment sets cover the query sequence. For each position in a query sequence (2gb1 in this example) there is a distinct set of 200 3-mer and a set of 200 9-mer fragments. This implies that the internal degrees of freedom Φ, Ψ, ω for any residue are restricted to a set of 2400 combinations. For three example positions, Φ, Ψ pairs are plotted on a Ramachandran map: THR located in a strand, LYS located in a helix and ASP located in a loop. Each red dot represents a Φ, Ψ pair from a fragment (2400 dots in each plot). Blue background in the maps shows the region allowed for the given amino acid type, computed from a non-redundant PDB subset with the BioShell package [10], [11].

Rosetta structure prediction protocols [1] generally begin with a low resolution coarse grained search of conformational space that uses a library of short peptide fragments (typically 3 and 9 residues long) as a Monte Carlo move set. The principle underlying fragment selection is that the set of conformations sampled by a particular short sequence is likely to be reasonably well approximated by the set of conformations that similar sequence segments sample in known protein structures. For each protein modeled, this library is selected from known structures based on the amino acid sequence and any other available information (see Fig. 1 for an illustrative example). The Rosetta nnmake program, written in Fortran, has been used up until now to pick fragments.

The extent to which the fragments recapitulate the actual local structure of a protein determines in part the overall success rate of structure prediction. The construction of the fragment libraries seeks to accurately represent not the most probable conformation for a given sequence segment, but the entire distribution of conformations the sequence segment is likely to adopt in protein structures. There is a tension between sampling too broadly (giving too diffuse a library) and sampling too narrowly (risking missing a critical set of torsion angles for a portion of the protein chain). The old NNMAKE program deals with this by generating libraries in which the frequencies of different secondary structural elements parallels that output by secondary structure prediction programs. Often there are non-congruent recommendations based on different “expert” scoring functions that incorporate different prior knowledge. Instead of using a single combined score, sets of the fragments derived from each “expert” scoring function are combined. Thus the resulting libraries represent a range of local structure conformations for each position. The NNMAKE architecture does not allow fine tuning of the tension in these objectives as needed for different applications, and is brittle when new prior information types are incorporated.

Local structural features such as kinks and bulges can result in bottlenecks for conformational sampling [2]. It is therefore crucial to start Rosetta modeling with the best possible fragments that incorporate all the available prior knowledge. Recent developments using experimental data to pick fragments, notably NMR chemical shift data (CS-ROSETTA), has shown that increases in fragment library quality can dramatically improve the quality of the resulting models. This has led to a demand for including many new types of information and data into the fragment picking process. Additionally, simultaneous design of sequence and structure calls for libraries that can vary during a search process. These evolving demands exceed the architectural limits of NNMAKE.

The new fragment picker described in this paper is modular and interactive to allow additional and evolving information content to more tightly focus libraries in a simple extensible manner. Here, we describe the new algorithm and demonstrate how it enables implementation of new Rosetta protocols. The fully customizable scoring function for fragment selection now allows use of any experimental data or prior knowledge. Throughout the text, ***bold italic font*** denotes fragment picking concepts while **typewriter font** is used to name the object classes as declared in Rosetta source code as well as names of files important to the picking process.

## Methods

The fragment library for a given protein is divided into sets for each position along the chain. The fragments at each position span a 3 or 9 residue window, which is overlapping with neighboring position windows. Thus to model a protein sequence composed of 100 amino acid residues, one needs 100−9 = 91 distinct sets of 9-mers and 97 sets of 3-mers. Typically each set comprises 200 fragments. Such a library contains 2400 possible triplets of Φ, Ψ, ω backbone internal coordinates for any given residue of a modeled protein.

**Figure 2:**
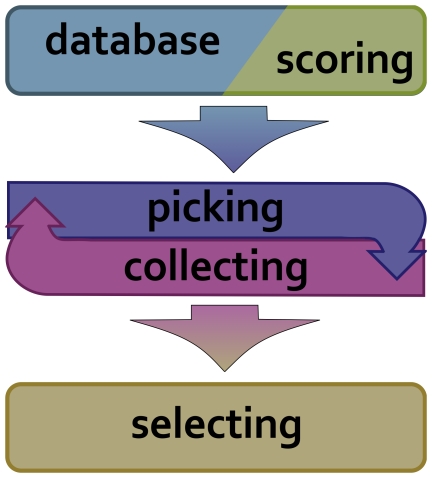
Overview of the fragment picking process. In the first step the program reads in a structure database file and creates the scoring system. During the second, iterative stage, each possible fragment i.e. a local match between a query sequence and a structure from the database is scored and sent to a candidates collector. In the last stage selector object picks the final fragment set based on the candidates gathered by the collector.

To generate the fragment libraries, the new fragment picker application reads a protein database file, a query sequence and few other necessary parameters and then writes a file, the fragment library, containing protein fragments of a desired length. The whole process can be split into three main steps (see. Fig. 2): (1) a fragment score function is created and database read in; (2) candidates are picked, scored and collected; and (3) final fragments are selected. The picking application is not only a stand-alone computer program, but also a collection of objects combined into a single pipeline. These parts can be modified or exchanged to match a specific task, such as the loop design protocol described below. The following section describes these fragment picking steps in context of the overall workflow. For brevity, let us denote the fragment length as L_F_, query sequence length as L_Q_ and a chunk (defined below) size as L_C_.

### Input data

**Figure 3:**
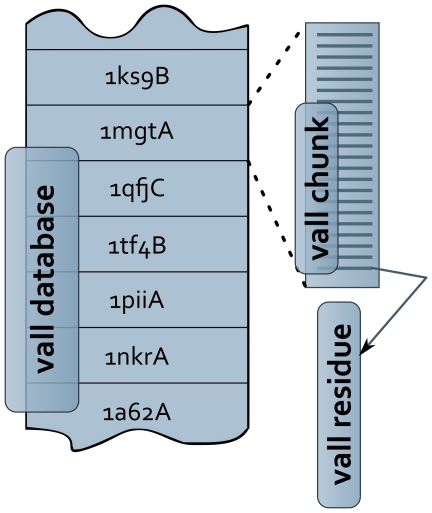
Organization of the structural database (nicknamed as vall). The database is divided into chunks and each chunk is composed of residues.

On startup, the application reads a number of input files. Some of them are mandatory and others are optional, depending on the chosen protocol, such as a quota definition file (see “Quota protocol” below) or on the scoring system, e.g. including chemical shifts or restraints. The mandatory input files are:

Query protein has to be defined by an amino acid sequence which can be specified by a sequence file in FASTA format (-in:file:fasta), a PsiBlast [3] sequence profile (-in:file:checkpoint), or by a protein structure in PDB format (-in:file:s).
Protein database file, nicknamed ***vall*** - is the source of all fragments (see. Fig. 3). Because Rosetta expects that each L_F_ -residue fragment is continuous and provides 3×L_F_ degrees of freedom, the selection process is limited to protein pieces with no chain breaks, referred as chunks (**VallChunk**). The current ***vall*** database is based on 9523 protein chains, on average a chunk has 245 residues but the range varies greatly from 5 to 1491 residues. Chunks that are shorter than a fragment length specified by a user are not taken into the picking process.
Scoring system configuration file has a tabular structure. Each line must contain at least four columns, that provide: (1) score term name, (2) score priority, (3) score weight, (4) maximum (worst) allowed score value. All the remaining fields in the line are treated as scoring term parameters and are passed to the relevant fragment score constructor. A detailed description of the scoring system is in the “Fragment scoring” section.

### Chunk-wise processing paradigm

After reading all the relevant input data, the application iterates the actual fragment picking by processing each **VallChunk** separately. Once a chunk has passed **VallChunkFilter** (e.g. **DenyPdbIdFilter** which is used by the protocols described in the next section), it is split into overlapping ***fragment candidates*** and each candidate is tested how it fits to any position in the query sequence. A fragment candidate is just a fragment-to-be; practically there is no difference between candidates and fragments that are written into an output file: fragments are just these candidates who survived the selection process. Once a fragment candidate has been scored, it is send to a ***fragment collector***. The procedure is repeated for every fragment size requested by a user. After that the next **vall** chunk is considered until the end of the **vall** database.

The chunk-wise processing assures that any **vall** chunk is processed only once. Moreover, all per-residue score components may be evaluated at once and stored in a L_Q_×L_C_ array. Many scores types are defined as a simple sum over all per-residue values along a fragment. In such a case these per-residue values are just read from a table without re-evaluating for overlapping fragments, which in some applications brings a remarkable efficiency gain. Unfortunately it also has a very serious drawback: we have to store all fragment candidates that are selected for all requested fragment sizes and all query sequence positions.

### Fragment scoring scheme

Total score **S** for a fragment is calculated as a linear combination of N_S_ score terms
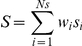
where the scores S_i_ and weights w_i_ are defined within the score configuration file (-frags::scoring::config). These scores describe a fragment's distance from the target, with lower values indicating a closer match. Whenever possible the individual score functions have been normalized such that a perfect match provides a score of 0 and complete failure to match provides a score of 1.

**Figure 4:**
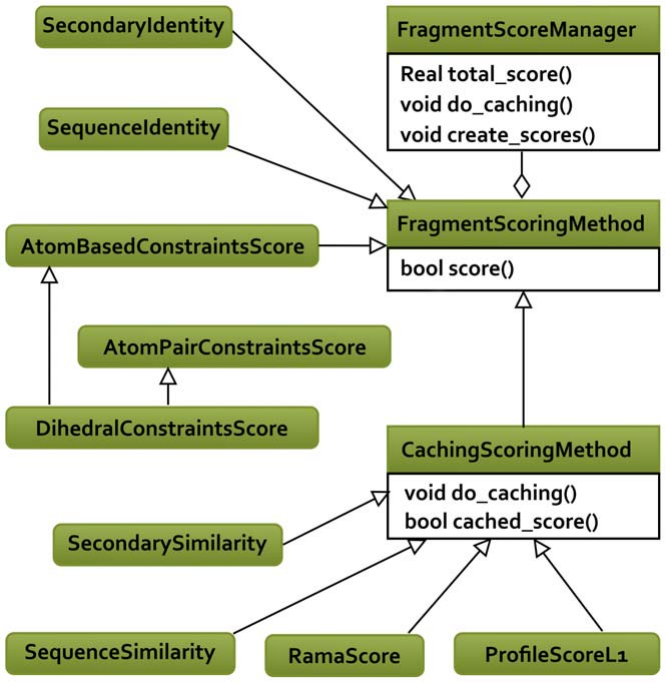
UML diagram showing the relations between score types. For the sake of clarity, only the base classes and the most commonly used score types are shown.

The object-oriented design for fragment scoring is similar to the Rosetta score function system. Its overall structure has been shown in the Fig. 4. Each score term S_i_ is a separate class, derived from the same **FragmentScoringMethod** base virtual class and have its own maker class. The **FragmentScoringManager** singleton implements a factory pattern [4]. It registers the makers, reads a file with score weights and creates the score function. The score values are held and passed around as **FragmentScoreMap** objects.

Often a given score-type is the sum of independent scores for each residue within the fragment. To avoid re-computing the residue scores that are shared between overlapping fragments the **CachingScoringMethod**, a class derived from **FragmentScoringMethod**, sets up a cache of residue scores prior to fragment scoring, filling a L_Q_×L_C_ matrix of per-position score components. Each fragment score is then calculated as a simple sum of cached residue comparisons.

The fragment picking system provides several score types, described in Table 1. The components S_i_ are evaluated in the order of decreasing priority, as assigned by the user. The user can also provide a maximum (worst) value allowed for each score component. If a given fragment exceeds that threshold, it is immediately discarded and all the remaining score components are not evaluated. In the most favorable situation, a restrictive score component that can be evaluated quickly may be used with the highest priority to avoid unnecessary calculations of the other score components. This speed-up is significant only in the case of non-caching scores; otherwise the full matrix of per-residue scores is evaluated before any fragment is considered from a current chunk.

**Table 1:** Most common score types for fragment assessment.

score type name	cacheable?	input files (file format)	comments
**SequenceIdentity**	no	amino acid sequence (FASTA, PDB)	counts amino acid types that are identical
**ProfileScoreL1**	yes	query sequence profile	L_1_ measure between amino acid probabilities
**SecondaryIdentity**	no	secondary structure prediction (psipred-SS2)	counts residues that have identical secondary structure
**SecondarySimilarity**	yes	secondary structure prediction (psipred-SS2)	L_1_ measure based on predicted seondary structure probabilities and the “true” observations as defined by DSSP
**CSScore**	yes	chemical shifts (TALOS)	
**RamaScore**	yes	secondary structure prediction (psipred-SS2)	based on Ramachandran map probabilities
**AtomPairConstraintsScore**	yes	distance restaints (Rosetta cst file)	any function based on the distance between any two atoms contained within a fragment
**DihedralConstraintsScore**	yes	dihedral restaints (Rosetta cst file)	any function based on the dihedral between any four atoms contained within a fragment
**FragmentCrmsd**	no	reference structure (PDB)	crmsd between a chunk fragment and a relevant part of a reference structure as a score

A brief list of most commonly used fragment score types and their required input files.

It should be stressed that a single score function will always attempt to saturate a fragment set with the winning option. For example, if a given residue has been predicted to have 51% chance to be in a helix and 49% chance to be in a strand then on its own the **SecondarySimilarity** score will select fragments that are helical at that position, even though a β-strand in this region is almost as likely as an α-helix. If the addition of other score types (**ProfileScoreL1**, **AtomPairConstraintsScore** or others) does not counterbalance this effect, the fragments may be highly biased toward some particular local geometry. To avoid this effect an optional quota mechanism can be used to select secondary structure elements according to the prediction rates.

### Collectors and selectors

PDB content is steadily growing and novel protein folds continue to be observed. This also causes the ***vall*** database to grow, which at its present size is large enough to create nearly 2 million fragment candidates (per query position). Storing all of these candidates in memory at the same time is not feasible, even for a moderate query protein size. Therefore, we provided the ***fragment collector*** object, which defines the rules for determining which fragments should be kept in memory as current candidates and which should be permanently rejected and removed from memory. This system uses a separate fragment collector for each user defined fragment size, and there are three main **CandidatesCollector** implementations provided in the class hierarchy:

**BoundedCollector** is based on a bounded priority queue. A bounded priority queue has a fixed pre-defined capacity and it keeps only the top-K candidates according to their final weighted scores. Inserting a good candidate forces the worst candidate to leave the queue. The priority is defined by a ***comparator*** object and can be customized by the user. The sorting order imposed by **BoundedCollector** is defined in a standard C++ way by a ***comparator*** object that provides strict weak ordering operator. The default behavior implemented in the picker is to compare total weighted scores of the two compared fragments, but user may easily customize it by passing a relevant object to a **BoundedCollector** constructor.
**GrabAllCollector** collects all the fragments that successfully passed the scoring stage. Its high memory usage makes it impractical for picking fragments for all the positions in a query sequence, but it might be very useful in some particular application, e.g. to enumerate and score all possible loop conformations that satisfy the score functions below a set of restrictive thresholds.
**QuotaCollector** is necessary when running a quota protocol (see the next section).

One should note that the collecting process accumulates the results during the chunk-wise ***vall*** processing. The decision whether to keep or to ignore a given candidate is based only on the fragments that were already processed. The program doesn't know anything about the candidates that will be collected from the downstream ***vall*** chunks that have not been assessed yet. In some applications a fragments final ranking depends upon the scoring properties of the ensemble of other fragments. To accomplish this without storing every fragment candidate, a compromise is to store the top N_C_ fragments according to a preliminary scoring criteria, derive the final ensemble-based criteria, and then select the top ranked subset, N_F_, of these.

The final choice of fragments is made by a **FragmentSelectingRule** object which returns N_F_ best fragments, based on all the N_C_ candidates stored in a collector. The most obvious selection algorithm is implemented in **BestTotalScoreSelector** class. In this case the content of a collector is sorted according to the total score and the best N_F_ candidates are reported as fragments. Obviously when N_C_ = N_F_, the selection process doesn't do anything; it just returns the content of the collector. Therefore in the cases where the selection stage is really necessary, the capacity of the collector N_C_ has to be much higher than N_F_. Users can easily implement their own selection rule to match their objectives.

### Quota mechanism

The quota system is a way to increase fragment diversity by defining a range of different fragment picking rule sets, then selecting the final fragments by taking a fixed number of fragments according to each set. This mechanism is used primarily for *ab-initio* protein structure prediction in order to diversify the fragments in two ways. First, by picking fragments according to three different secondary structure prediction “experts”, with each prediction being used to select its own independent set of fragments, and second, by selecting the final fragments such that the secondary structure frequency in the fragment population is approximately the same as the predicted propensity.

Although the quota mechanism has only been applied for diversifying secondary structure, its design is intended to be as general as possible. One can diversify fragments based on any property or observable providing that it can be computed both for the query sequence and for each protein stored in the ***vall***. The concepts and components of the quota system are:

- **QuotaPool** is a container that collects fragments for only one specific feature, e.g. only these fragments where the middle residue is helical.
- ***quota allowance*** says what fraction of the total number of candidates N_C_ will be collected by a given ***quota pool***; a **QuotaPool** class implementation is based on a **BoundedCollector**.
- **QuotaCollector**- a specialized implementation of a **CandidatesCollector** type where fragment candidates are stored in quota pools separately for each category of the diversified observable.
- **QuotaSelector** - is aware of the internal structure of a **QuotaCollector** and selects the final fragments matching the predefined quota allowance fractions.

The manner a given **QuotaPool** operates is defined by its two distinct and, in principle, independent features: (1) a “hard” rule that decides whether a given fragment candidate is accepted or denied by a pool, and (2) scoring scheme that is used to score fragments within the pool. These two will be described here on the example of secondary structure quota implementation. The strict acceptance rule in this case is based on a middle residue of a candidate. Each **SecondaryStructurePool** object is constructed to accept only one secondary structure type: either H, E or L. The “true” secondary structure classification for the candidates has been defined with DSSP program and is stored in the ***vall*** database.

In the simplest case, secondary structure based quota consist of three pools; each of them collects one of the three secondary structure types (by the means of the acceptance rule, as mentioned above). User may arbitrarily decide quota allowance shares for the pools, effectively changing the relative abundance of each secondary structure type in the final set of fragments. Following the algorithm implemented in the nnmake program, the quota protocol assigns the quota allowance values based on the predicted probability for each secondary structure type at a given position. In the case when the quota is based on a single secondary prediction, the three pools share the same scoring system. In a more advanced protocol (which is actually used for *ab-initio* structure prediction), described in the “Quota protocol” section, three secondary structure predictors are used and the pools differ in their scoring scheme.

### Protocols

This section provides a detailed description of two protocols that can be accessed by calling **picker** application with proper flags. All these protocols rely on query sequence profile, on the Rosetta database directory and the ***vall*** file. The program also reads a flag-file and a score configuration file, although the content of the two is protocol-dependent.

#### Best fragments protocol

This very basic example illustrates how to pick the best fragments according to a sequence profile and a secondary structure. The example is not intended to be a working protocol. It is rather an illustration of how to declare the basic elements of the fragment picking system. The example shows also several useful features that have been introduced with the new picker. The whole protocol is in fact a single command:

picker.linuxgccrelease @best-fragments-protocol.flags

The flags that control the fragment picking process are:

**# Input databases**

**-database ../database**

**-in:file:vall ../vall.apr24.2008.extended.gz**

**# Query-related input files**

**-in::file::checkpoint input_files/2jsvX.checkpoint**

**-in::file::s input_files/2jsvX.pdb**

**-frags::ss_pred input_files/2jsvX.psipred.ss2 predA**

**# Weights file**

**-frags::scoring::config input_files/simple.wghts**

**# What should we do?**

**-frags::bounded_protocol**

**# three-mers only, please**

**-frags::frag_sizes 3**

**-frags::n_candidates 200**

**-frags::n_frags 200**

**# Output**

**-out::file::frag_prefix output_files/frags**

**-frags::describe_fragments output_files/frags.fsc**

The flags provide the necessary input databases, and input files: score weights, sequence profile (2jsvX.checkpoint), reference structure and secondary structure prediction. The secondary structure prediction flag is given a tag-name by an arbitrary string identifier, in this case **predA**. The -frags::bounded_protocol flag sets up a **BoundedCollector** and a **BestTotalScoreSelector**. Further options define the desired fragment set, which in this case should contain two hundred 3-mers for each residue. The fragments will be selected from the collector with 200 candidates, which means that the final selection step doesn't do anything here, just returns all the collected candidates as fragments. Finally, the two last flags define the name of the output fragment file and fragment score file (described below).

The score weight file:

**# score name priority wght max extras**

**RamaScore 400 2.0 - predA**

**SecondarySimilarity 350 1.0 - predA**

**ProfileScoreL1 200 0.5 -**

**FragmentCrmsd 0 0.0 -**

defines three score components. One can find the “**predA**” tag here, which connects the secondary structure prediction file (2jsvX.psipred.ss2 in above example) with its **SecondarySimilarity** score. In general, users can apply several **SecondarySimilarity** scores at a time, each of them based on its own secondary structure prediction and with a different weight, e.g. reflecting *a priori* knowledge about the accuracy of these predictors. The tag system is the only way to connect a secondary structure prediction file with the proper score component. The secondary structure prediction is also necessary for **RamaScore** score, which forces the candidates to lay in the allowed region of the relevant Ramachandran map. **FragmentCrmsd** score has weight 0.0 and thus it does not affect the total score and has no influence on fragment picking. Scores with zero-weights will have their values printed into a fragment score file and thus are useful as metrics. In this case **FragmentCrmsd** is used to provide the Cα-RMSD of each fragment when superimposed onto the appropriate window of the user defined target pdb (-in:file:s), allowing for measurement of the fragments' quality given the structure of a known target. Such zero-weighted scores are termed as ***late scoring*** in the source code; the program evaluates them only for the final fragments and they have practically no impact on the execution time.

The fragment score file is produced only when -frags::describe_fragments flag is given. There will be a separate file for each fragment size. Each line of the file describe a single fragment, providing:

- starting position in a query sequence
- starting position in a source protein, as recorded in the vall database
- PDB id of the source protein
- chain id of the source protein
- secondary structure for the middle residue in the fragment
- values of all the score components, ordered according to the descending score priority
- total weighted score
- fragment id, which is the line number in the vall file where the fragment starts (provided for debugging purposes)

#query_pos vall_pos pdbid c ss RamaScore 

1 147 2h2z A E 0.00

1 2 2qg8 A E 0.00

1 154 1zbp A E 0.00

1 128 2r0x A E 0.00

1 101 2v1l A E 0.00

SecondarySimilarity FragmentCrmsd TOTAL FRAG_ID

0.16 0.34 0.496 1789106

0.16 0.44 0.496 2169849

0.16 0.28 0.496 1327285

0.16 0.31 0.496 2204322

0.16 0.31 0.496 2243744

#### Quota protocol

The second example demonstrates how to pick fragments for an *ab-initio* structure prediction task, where fragments are diversified to preserve the secondary structure type which, according to predictions, is less probable. Because individual secondary structure predictions can often mispredict, the protocol takes into account the predictions made by three independent “expert” programs: PsiPred [5], Jufo and SAM [6], which results in a total of 9 quota pools (three secondary structure propensity pools (H, E and L) for each of the three predictors). Quota allowance for each pool is computed as the product of the predictor allowance and the secondary structure type propensity as predicted by this predictor. Predictor allowance fractions are defined in **quota.def** file:

**#pool_id pool_name fraction**

**1 psipred 0.6**

**2 jufo 0.2**

**3 sam 0.2**

The score weight file looks in this case as follows:

**# score name priority wght max extras**

**SecondarySimilarity 350 0.5 - psipred**

**SecondarySimilarity 300 0.5 - sam**

**SecondarySimilarity 250 0.5 - jufo**

**RamaScore 150 1.0 - psipred**

**RamaScore 150 1.0 - jufo**

**RamaScore 150 1.0 - sam**

**ProfileScoreL1 200 1.0 -**

**FragmentCrmsd 30 0.0 -**

and provides three **SecondarySimilarity** score components based on three different predictors. Note, that the tags assigned to score components match tags in the **quota.def** file. Finally, the following flag file is used:

**# Input databases**

**-database ../database**

**-in:file:vall ../vall. apr24.2008.extended.gz**

**# Weights file**

**-frags::scoring::config input_files/quota_protocol.wghts**

**# Query-related input files**

**-in::file::checkpoint input_files/2jsvX.checkpoint**

**-in::file::s input_files/2jsvX.pdb**

**-frags::ss_pred input_files/2jsvX.psipred.ss2 psipred input_files/2jsvX.sam.ss2 sam input_files/2jsvX.jufo.ss2 jufo**

**# Get rid of homologues fragments**

**-frags::denied_pdb input_files/2jsvX.homolog_vall**

**# Quota.def file defines the shares between different quota pools.**

**# The total should be 1.0**

**-frags::picking::quota_config_file input_files/quota.def**

**# we need nine-mers and three-mers**

**-frags::frag_sizes 9 3**

**# Select 200 fragments from 700 candidates. We need more candidates**

**# than fragments for the selector to work properly**

**-frags::n_candidates 700**

**-frags::n_frags 200**

**# Output**

**-out::file::frag_prefix output_files/frags**

**-frags::describe_fragments output_files/frags.fsc**

**Figure 5:**
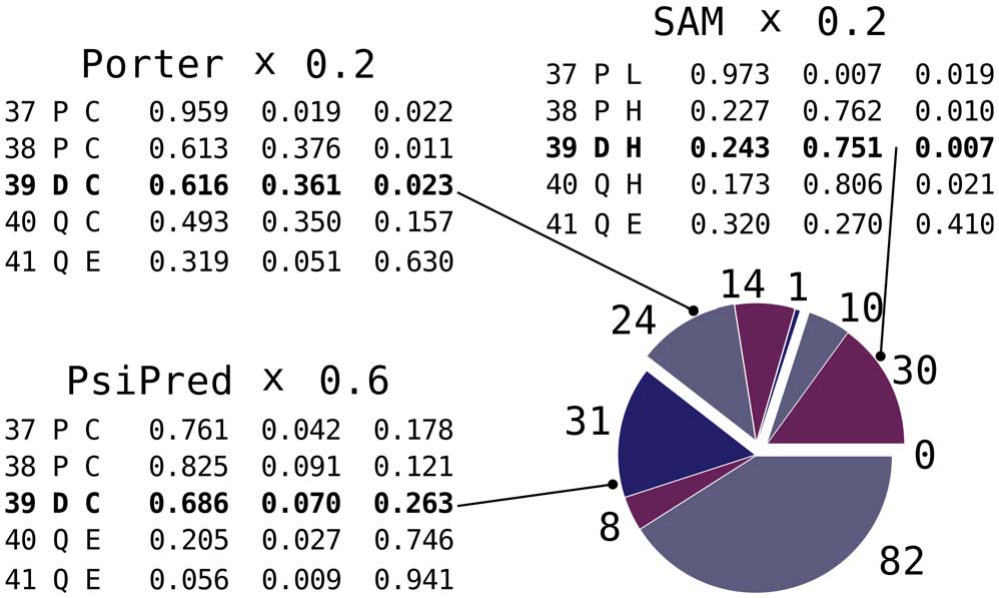
Quota example: the number of fragments assigned to each quota pool based on actual prediction for the sequence of Ubiquitin, residue 39. There are nine pools, based on three secondary structure predictors (PsiPred, Jufo and SAM) predicting the three secondary structure types: helical (H, purple), coil (C, gray) and extended (E, blue). The order of columns with predicted probabilities is: C, E, H. Notice, that SAM predicted coil while PsiPred and Jufo a helix. While PsiPred's prediction however says “C with E possible”, Jufo gives a slight chance to H. The ninth pool (E for SAM) has size 0 in this case.

In addition to the flags known from the previous example, the flag file provides also a quota definition file (**quota.def**) and asks both for 3-mers and 9-mers. The three secondary structure prediction files are given one by one with their string identifiers in a single line. The **-frags::denied_pdb** flag initiates **DenyPdbIdFilter** object which filters out unwanted chunks based on their PDB id and in fact is not the part of the protocol in its real-life application. The flag has been specified here to avoid contaminating the resulting fragment set by fragments coming from homologous structures, as in practice the *ab-initio* protocol is typically only used when no homologous structures are available. Figure 5 gives a real-life illustration of the quota system, depicting the nine quota pools in three groups (PsiPred, SAM and Jufo), each with three pools. The three groups are based on which secondary structure prediction will be used to score the fragments that group selects, and each groups associated pools differ based on the secondary structure allowed at the middle residue, either E, H, or L. In other words, the pools within a group share the same secondary structure similarity score function but are targeted for different secondary structure type. The three score functions contain the same scoring terms: **ProfileScoreL1**, **SecondarySimilarity** and **RamaScore**. The latter two however depend on predicted secondary structure probabilities, which results in different score values for the same candidate.

Note also, that in this case N_C_ is much higher than N_F_. This is necessary to provide enough candidates for the selection step in order to get assumed number of fragments. To explain this detail let us assume that all the three predictors gave 10% chance to a helix at a certain position. Taking into account the shares of the three predictors defined as in **quota.def** file listed above, one would expect 200 * 0.1 = 20 helical fragments: 12 from PsiPred, 4 from SAM and 4 from Jufo. During the collecting process each candidate is offered to every pool; a helical candidate may be accepted by any of the three helix-oriented quota pools. If the scoring functions assigned to pools are very similar to each other (i.e. when the three secondary structure predictions are very similar), the three collectors may contain the same candidates, which in the worst-case yields only 12 distinct fragments.

#### Flexible loop design protocol

A critical improvement provided by the new fragment picker is the ability to update the selected fragments when the protein sequence is perturbed, as is the case for an iterative protein design process. A Rosetta protein design process that allows a flexible backbone will typically alternate between structure prediction and sequence perturbation for many cycles. Accurate predictions of designed structures elevates confidence in the resulting models. This continuously focusing design cycle method has enabled the creation of a novel protein topology [7]. Conventionally, fragment libraries are static during this optimization because they are generated beforehand by NNMAKE. Since the design sequence is not known beforehand, these static libraries are based either on the starting sequence or using a generic poly-Ala/poly-Val sequence. The new fragment picker allows the fragment library to update on-the-fly as the sequence changes. A simplified *ad hoc* version of this concept had been previously implemented in a branch of Rosetta; this new version however couples the full power and configurability of the fragment picker into Rosetta.

**Figure 6:**
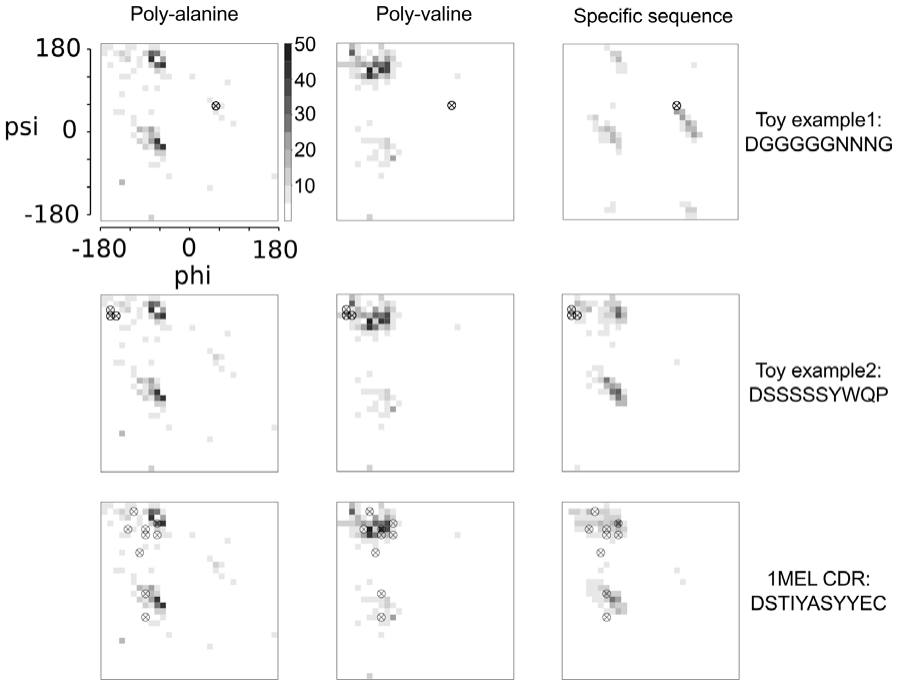
Phi/Psi distributions of picked fragments using different query sequences and structures. Each row represents a different target loop structure. Each column is a different method for deriving the fragment sets: poly alanine, poly valine, and a structure-specific sequence. Encircled crosses in each figure show the phi-psi of the prepared fragment (input structure). Each square represents a phi/psi bin, where the color reflects the number of phi/psi values for the middle residue of the selected fragments. The fragment distribution picked using a specific sequence is variable and has density most consistent with the input backbone structure (encircled crosses).

In theory, having fragments that correspond directly to the current sequence in a simulation should result in superior performance. As evidence that specific sequences select fragments more structurally similar to the input, we constructed a simple fragment selection test. We selected fragments from three loop structures with different input sequences (poly-alanine, poly-valine or a sequence taken from the structure). Two of the loop structures were artificially fabricated by setting each Φ, Ψ to Ramachandran pairs that were rare but plausible, then repacked with Rosetta to determine the optimal sequence. By construction, these “toy” loops anticipate structures where poly-alanine and poly-valine input sequences will under-sample the proper fragments. A third test case used a natural loop from an antibody (1MEL) where we can expect that subtle details in the structure could matter to function. The fragments selected using the structure-specific sequence have distinctly focused Φ, Ψ distributions that are better reflective of the prepared structure (Figure 6).

To demonstrate how the fragment picker components can be modified to achieve a project-specific goal, here we present a simple protocol for designing loops. The protocol updates the set of fragments throughout the simulation. This design-mode fragment picking requires two features: 1) there must be no dependence on pre-computed data, because we must compute on-the-fly and 2) a direct API must exist between the fragment picker objects (e.g. **FragmentPicker**) and the fragment mover objects (e.g. **ClassicFragmentMover**). The two most common sequence-dependent input data for fragment picking protocols are a secondary structure objective and a sequence profile. In our loop design application the secondary structure prediction is simple: everything is in a loop conformation. For a sequence profile we have a choice of supplying the exact sequence of the current design iteration step, or somehow generating a broader sequence profile. In this example, we generate a profile by convolving the current sequence with a BLOSUM62 sequence substitution matrix. A more complex protocol might use convolution conditioned on a preferred secondary structure or might up-weight the exact sequence more as the design process converged. We created a fragment score type (**ProfileScoreSubMatrix**), which generates a sequence profile using just the sequence in the current pose. Finally, the new fragment picker to fragment mover API utilizes the **ConstantLengthFragSet** object and converts the fragment residues (**VallResidue**) into fragment frames (**Frame** and **AnnotatedFragData** objects).

The protocol can be run with:

flexibleLoopDesign.linuxgccrelease @flex-loop-protocol.flags

and the flex-loop-protocol.flags flag file looks as follows:

**# Input structure to design**

**-in:file:pdb input_files/flexloop_pdb.pdb**

**# Input databases**

**-database ../database**

**-in:file:vall ../vall.apr24.2008.extended.gz**

**# Weights file**

**-frags::scoring::config input_files/flexloop_protocol.wghts**

**# Residue file for which residue will be designed**

**-resfile input_files/flexloop_resfile.txt**

**# Loop defintion file**

**-loops:loop_file input_files/flexloop_loopdef.txt**

**# Run Structure prediction/Structure design cycle 10 times**

**-dwkulp::nSteps 10**

**# Constant seed for protocol demo**

**-run::constant_seed**

The protocol flags are similar to previous protocols, except here we add standard Rosetta res and loop files and a starting protein structure as design parameters.

The scoring scheme is as follows:

**#_score_name priority wght max extras**

**SecondarySimilarity 350 2.0 - loop**

**ProfileScoreSubMatrix 200 1.0 - BLOSUM62.txt**

The scoring configuration file has the same format as previous examples. The **flexibleLoopDesign** program requires that the **SecondarySimilarity** scorer be passed the extra flag “loop”. The flag for the **ProfileScoreSubMatrix** scorer is the full path and filename of an amino acid substitution matrix file, where each natural amino acid gets a score if it were to change into each of the 19 other natural amino acids. Here, we used a BLOSUM62 matrix for this purpose.

### Modifying Protocols

The main advantage of the new system is that a user can easily alter the fragment picking process by simple adjustments to its configuration files. Here we provide two illustrative examples.

#### Using restraints

It is possible to use restrains during fragment picking in a similar way as they are used in Rosetta runs. Currently two kinds of Rosetta restraints (for legacy reasons they are referred in Rosetta as “constraints”) are accessible from the picker:

**AtomPairConstraintsScore** and **DihedralConstraintsScore**. The former type of a restraint is used to favor a certain distance value between two desired atoms within a fragment. The latter score is based on four arbitrary atoms and it restraints a dihedral angle. During fragment picking restraints may be applied only to Cβ and backbone atoms as all other atoms are not available from the vall database. Moreover, to be used the restraints must be contained within the fragment size, e. g. when nine-mers are to be selected based on **AtomPairConstraint** score between atoms from i-th and j-th residue, the condition |i−j|<9 must be satisfied. Inappropriate restraints are ignored by the program, with a warning message printed while reading the data file.

To pick restrained fragments one has to use the -constraints::cst_file flag to provide a file with restraints data and add the **AtomPairConstraintsScore** term to the score weight file. The standard Rosetta file format is used for restraints data, an example is provided along with the protocol capture. As with any fragment scoring term, restraints are protocol independent and can be combined with any of the examples shown. Here we illustrate this feature using TEDOR data (Solid-State NMR measurement) collected for GB1 protein [8]. Distance restraints in XPlor format have been downloaded from a BMRB database [9], filtered and converted to Rosetta *.cst file format. All the input files, command line and the output may be found in the protocol capture data.

#### Using torsion class score

The second modification example shows the use of **TorsionBinSimilarity** score, which is based on 5-state torsion angle class definition [2]. The five states, denoted by five ABEGO letters represent different regions in Ramachandran space and a cis-omega conformation (bin O). User should use -in::file::torsion_bin_probs flag to introduce an input file that provides probability of finding each of the states at a given position in an amino acid sequence. The score term assesses the match between the provided probabilities and the actual conformation observed for a ***vall*** residue. The input probabilities, for instance, may be obtained by a machine learning method or extracted from template structures that are used for modeling.

In this example we tweak the input file to selectively introduce a bulge at a single position in a β-strand while leaving all the other positions unaffected. For the sake of simplicity, we demonstrate this feature along with the “BestCandidates” protocol. The necessary input file (input_files/2jsvX.abego in this case - it may be found in the protocol capture data) consists of seven columns: residue number, torsion class ID and the five probabilities given in the ABEGO order. In this example all the probabilities are equal to 1.0 except the position 16, which we decided to alter. The bulge is enforced by favoring bin ‘A’ in the middle of a strand. Torsion bin probability 1.0 results in the lowest possible score 0.0, probability 0.0 on the contrary gives (the highest) score 1.0. Thus for the rows when all the probabilities are set to 1.0 the score is always 0.0 and in these cases **TorsionBinSimilarity** does not affect the selection process. At the position 16 only the conformations with α bin get score 0 and all the other are penalized with the highest possible value.

Besides the change in a command line, a **TorsionBinSimilarity** score term must be added to a score weight file along with appropriate parameters. Here we used:

**TorsionBinSimilarity 500 1.0 -**

The resulting fragments are basically the same as for the “BestCandidates” case. The only difference is that all fragments (both 3-mers and 9-mers) that cover the residue 16 include a bulge. Obviously this bulge will also be present in structures calculated in a Rosetta run that is based on these fragments. One can introduce a kink into a helix in the same way, by forcing a residue to be of “E” class.

## Results

**Figure 7:**
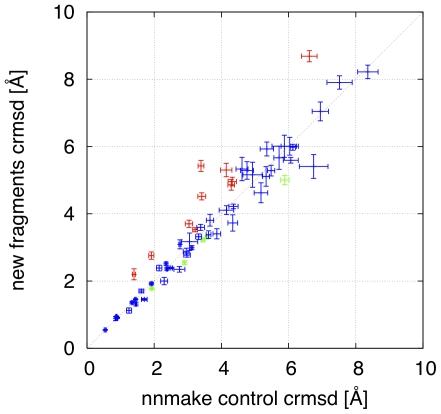
*Ab-initio* benchmark: each symbol corresponds to a single protein target, for which an *ab-initio* structure prediction has been run with the reference and the new fragments (X an Y axis, respectively). Red (green) points denote targets for the new algorithm yields worse (better) results. For targets marked by blue symbols no significant difference has been observed.

The design on the new fragment picker is a tradeoff between efficiency and flexibility. Modular, object oriented design leads to unavoidable overheads. Nevertheless run time of the new application is comparable to nnmake, and the order of the new algorithm is linear as a function of the query protein size. Although we put a lot of effort into recapturing the original behavior of nnmake, subtle differences between the algorithms and in secondary structure handling result in different fragment sets. To provide an ultimate comparison between the two fragment picking applications, we ran an *ab-initio* prediction on a benchmark set of 62 proteins (for the list of targets see Table S1). The two groups of folding simulations were based on two fragment sets: the *reference* one, obtained by nnmake with standard settings and the *new* one, derived as described in “Quota protocol” section above. The benchmark has been performed by the means of Rosetta@home distributed computing project. We spent 500 work units for each target, which resulted in around two CPU-months (roughly 1400 CPU-hours) of calculations per target, both for the control run as well as for the folding simulations with the new fragments. Because the target sequences vary in the number of amino acids, different targets yielded different number of models, ranging from 4065 for 1cg5B (141 residues) to 19183 for 1pgxA (55 residues). Figure 7 shows 0.1-percentile crmsd obtained from simulations with the new fragments (Y axis) as a function of the same quantity based on the reference fragments (X-axis). Each symbol in the figure represents a single target protein. The crmsd 0.1-percentiles (the point location) and their standard deviation (marked as error bars) were computed by 50-fold bootstrap procedure. The plot shows that the two fragment sets are equivalent in quality, as they result in very similar protein predictions, both for successful targets and for failures.

## Discussion

We have developed a new algorithm for selecting protein fragments used by Rosetta for protein structure modeling. The new fragment picking system is quite comparable to the old one in terms of the results of *ab-initio* structure prediction benchmark. The big advantage of the new method is that it is object oriented and additional scoring terms, representing for example new experimental data sources, can readily be incorporated. The user can fine-tune the picking process through the **FragmentScoreManager** and the **BoundedCollector**. The new picker has been specifically designed to incorporate new kinds of data as easily as possible. The program as well as all the files necessary to run the example calculations described in this contribution has been made publicly available with Rosetta 3.3 version, released on July 26, 2011.

## Supporting Information

Table S1 **Comparison between nnmake program and the new fragment picker.** The *ab-initio* benchmark set comprises 62 small globular proteins. For each target, coordinate root-mean square deviation (crmsd) of the top 0.1% model is reported based on extensive Rosetta computations. Columns 7, 8: the reference (nnmake) fragments, columns 9, 10: fragments selected by the new algorithm; avg and sdev are mean and standard deviation from 50-fold 0.1 percentile bootstrap estimation (see also Figure 6).
(DOC)

## References

[1] Leaver-Fay A, Tyka M, Lewis SM, Lange OF, Thompson J (2011). ROSETTA3: an object-oriented software suite for the simulation and design of macromolecules.. Methods Enzymol.

[2] Kim DE, Blum B, Bradley P, Baker D (2009). Sampling bottlenecks in de novo protein structure prediction.. J Mol Biol.

[3] Altschul SF, Madden TL, Schaffer AA, Zhang J, Zhang Z (1997). Gapped BLAST and PSI-BLAST: a new generation of protein database search programs.. Nucleic Acids Res.

[4] Gamma E, Helm R, Johnson RE, Vlissides J (2005). Design patterns: elements of reusable object-oriented software.

[5] Ward JJ, McGuffin LJ, Buxton BF, Jones DT (2003). Secondary structure prediction with support vector machines.. Bioinformatics.

[6] Karplus K (2009). SAM-T08, HMM-based protein structure prediction.. Nucleic Acids Res.

[7] Kuhlman B, Dantas G, Ireton GC, Varani G, Stoddard BL (2003). Design of a novel globular protein fold with atomic-level accuracy.. Science.

[8] Nieuwkoop AJ, Wylie BJ, Franks WT, Shah GJ, Rienstra CM (2009). Atomic resolution protein structure determination by three-dimensional transferred echo double resonance solid-state nuclear magnetic resonance spectroscopy.. J Chem Phys.

[9] Doreleijers JF, Vranken WF, Schulte C, Lin J, Wedell JR (2009). The NMR restraints grid at BMRB for 5,266 protein and nucleic acid PDB entries.. J Biomol NMR.

[10] Gront D, Kolinski A (2008). Utility library for structural bioinformatics.. Bioinformatics.

[11] Gront D, Kolinski A (2006). BioShell–a package of tools for structural biology computations.. Bioinformatics.

