# Supplementary material for: Generalized Fragment Picking in Rosetta: Design, Protocols and Applications
Source: PLoS One. 2011 Aug 24;6(8):e23294. doi: 10.1371/journal.pone.0023294 (PMC3160850; doi:10.1371/journal.pone.0023294)
Supplement: Table S1 — Comparison between nnmake program and the new fragment picker. The ab-initio benchmark set comprises 62 small globular proteins. For each target, coordinate root-mean square deviation (crmsd) of the top 0.1% model is reported based on extensive Rosetta computations. Columns 7, 8: the reference (nnmake) fragments, columns 9, 10: fragments selected by the new algorithm; avg and sdev are mean and standard deviation from 50-fold 0.1 percentile bootstrap estimation (see also Figure 6). (DOC) [file pone.0023294.s001.doc]

**Table S1.** Comparison between nnmake program and the new fragment picker.

| **target** | | | | | | nnmake result | | picker result | |
| --- | --- | --- | --- | --- | --- | --- | --- | --- | --- |
| **PDB** | **chain** | from | to | nres | SCOP class | avg | sdev | avg | sdev |
| **1a19** | **A** | 1 | 89 | 89 | c.9.1.1 | 3.03 | 0.12 | 3.07 | 0.14 |
| **1a32** | **A** | 21 | 85 | 65 | a.16.1.2 | 0.92 | 0.01 | 0.90 | 0.02 |
| **1a68** | **A** | 1 | 87 | 87 | d.42.1.2 | 5.29 | 0.22 | 5.53 | 0.17 |
| **1acf** | **A** | 1 | 125 | 125 | d.110.1.1 | 3.85 | 0.17 | 4.31 | 0.15 |
| **1ail** | **A** | 1 | 70 | 70 | a.16.1.1 | 4.16 | 0.07 | 4.36 | 0.15 |
| **1aiu** | **A** | 1 | 105 | 105 | c.47.1.1 | 1.43 | 0.04 | 1.39 | 0.02 |
| **1b3a** | **A** | 13 | 67 | 55 | d.9.1.1 | 1.93 | 0.05 | 1.69 | 0.03 |
| **1bgf** | **A** | 3 | 120 | 118 | a.90.1.1 | 5.07 | 0.21 | 5.42 | 0.23 |
| **1bk2** | **A** | 1 | 57 | 57 | b.34.2.1 | 3.71 | 0.09 | 3.58 | 0.06 |
| **1bkr** | **A** | 1 | 108 | 108 | a.40.1.1 | 5.90 | 0.23 | 5.92 | 0.11 |
| **1bm8** | **A** | 1 | 99 | 99 | d.34.1.1 | 3.52 | 0.13 | 3.12 | 0.05 |
| **1bq9** | **A** | 2 | 52 | 51 | g.41.5.1 | 2.94 | 0.09 | 2.64 | 0.07 |
| **1c8c** | **A** | 1 | 62 | 62 | b.34.13.1 | 1.70 | 0.05 | 1.43 | 0.04 |
| **1c9o** | **A** | 1 | 66 | 66 | b.40.4.5 | 2.85 | 0.05 | 2.91 | 0.06 |
| **1cc8** | **A** | 1 | 72 | 72 | d.58.17.1 | 2.20 | 0.09 | 2.20 | 0.08 |
| **1cei** | **A** | 1 | 85 | 85 | a.28.2.1 | 6.02 | 0.20 | 5.61 | 0.12 |
| **1cg5** | **B** | 1 | 141 | 141 | a.1.1.2 | 5.83 | 0.13 | 5.54 | 0.23 |
| **1ctf** | **A** | 1 | 68 | 68 | d.45.1.1 | 3.11 | 0.06 | 2.96 | 0.10 |
| **1dhn** | **A** | 1 | 121 | 121 | d.96.1.3 | 6.86 | 0.30 | 7.02 | 0.13 |
| **1e6i** | **A** | 1 | 110 | 110 | a.29.2.1 | 5.07 | 0.20 | 4.70 | 0.18 |
| **1elw** | **A** | 1 | 117 | 117 | a.118.8.1 | 0.55 | 0.01 | 0.54 | 0.01 |
| **1enh** | **A** | 1 | 54 | 54 | a.4.1.1 | 1.73 | 0.05 | 1.61 | 0.07 |
| **1ew4** | **A** | 1 | 106 | 106 | d.82.2.1 | 5.64 | 0.12 | 5.62 | 0.13 |
| **1eyv** | **A** | 1 | 131 | 131 | a.79.1.1 | 4.97 | 0.12 | 5.00 | 0.33 |
| **1fkb** | **A** | 1 | 107 | 107 | d.26.1.1 | 8.31 | 0.12 | 8.61 | 0.23 |
| **1fna** | **A** | 1 | 91 | 91 | b.1.2.1 | 3.70 | 0.12 | 4.25 | 0.09 |
| **1gvp** | **A** | 1 | 87 | 87 | b.40.4.7 | 5.82 | 0.07 | 5.80 | 0.09 |
| **1hz6** | **A** | 7 | 67 | 61 | d.15.7.1 | 1.94 | 0.03 | 2.35 | 0.02 |
| **1ig5** | **A** | 1 | 75 | 75 | a.39.1.1 | 2.49 | 0.03 | 2.42 | 0.03 |
| **1iib** | **A** | 1 | 103 | 103 | c.44.2.1 | 2.81 | 0.08 | 2.60 | 0.11 |
| **1kpe** | **A** | 1 | 108 | 108 | d.13.1.1 | 4.73 | 0.14 | 4.63 | 0.17 |
| **1lis** | **A** | 7 | 131 | 125 | a.19.1.1 | 6.22 | 0.20 | 5.73 | 0.21 |
| **1lou** | **A** | 1 | 92 | 92 | d.58.14.1 | 4.32 | 0.06 | 4.85 | 0.09 |
| **1nps** | **A** | 1 | 88 | 88 | b.11.1.1 | 4.52 | 0.14 | 4.91 | 0.09 |
| **1opd** | **A** | 1 | 85 | 85 | d.94.1.1 | 3.14 | 0.06 | 3.24 | 0.04 |
| **1pgx** | **A** | 8 | 62 | 55 | d.15.7.1 | 0.87 | 0.02 | 0.89 | 0.02 |
| **1ptq** | **A** | 1 | 50 | 50 | g.49.1.1 | 5.39 | 0.37 | 5.87 | 0.19 |
| **1r69** | **A** | 1 | 63 | 63 | a.35.1.2 | 1.43 | 0.02 | 1.52 | 0.04 |
| **1rnb** | **A** | 1 | 109 | 109 | d.1.1.2 | 7.61 | 0.28 | 8.00 | 0.15 |
| **1scj** | **B** | 1 | 66 | 66 | d.58.3.2 | 2.52 | 0.03 | 2.43 | 0.01 |
| **1shf** | **A** | 1 | 59 | 59 | b.34.2.1 | 3.40 | 0.04 | 3.23 | 0.05 |
| **1ten** | **A** | 2 | 90 | 89 | b.1.2.1 | 2.89 | 0.11 | 2.09 | 0.10 |
| **1tif** | **A** | 1 | 59 | 59 | d.15.8.1 | 2.38 | 0.04 | 2.72 | 0.03 |
| **1tig** | **A** | 1 | 88 | 88 | d.68.1.1 | 2.98 | 0.09 | 2.85 | 0.03 |
| **1tit** | **A** | 1 | 89 | 89 | b.1.1.4 | 2.65 | 0.06 | 2.24 | 0.05 |
| **1tul** | **A** | 1 | 102 | 102 | b.85.5.1 | 8.73 | 0.18 | 7.89 | 0.62 |
| **1ubi** | **A** | 1 | 76 | 76 | d.15.1.1 | 2.29 | 0.15 | 2.05 | 0.03 |
| **1ugh** | **I** | 1 | 82 | 82 | d.17.5.1 | 4.54 | 0.09 | 4.15 | 0.08 |
| **1urn** | **A** | 7 | 96 | 90 | d.58.7.1 | 3.22 | 0.07 | 3.56 | 0.07 |
| **1utg** | **A** | 1 | 70 | 70 | a.101.1.1 | 3.52 | 0.06 | 3.34 | 0.07 |
| **1vcc** | **A** | 1 | 77 | 77 | d.121.1.1 | 3.62 | 0.07 | 3.28 | 0.04 |
| **1vie** | **A** | 4 | 59 | 56 | b.34.4.1 | 4.05 | 0.12 | 4.36 | 0.14 |
| **1who** | **A** | 1 | 94 | 94 | b.7.3.1 | 5.30 | 0.37 | 5.20 | 0.41 |
| **1wit** | **A** | 1 | 93 | 93 | b.1.1.4 | 4.33 | 0.17 | 3.83 | 0.15 |
| **256b** | **A** | 1 | 106 | 106 | a.24.3.1 | 1.42 | 0.05 | 1.39 | 0.05 |
| **2acy** | **A** | 1 | 98 | 98 | d.58.10.1 | 5.70 | 0.28 | 5.68 | 0.22 |
| **2chf** | **A** | 1 | 128 | 128 | c.23.1.1 | 2.42 | 0.13 | 2.70 | 0.08 |
| **2ci2** | **I** | 4 | 65 | 62 | d.40.1.1 | 2.96 | 0.13 | 3.54 | 0.08 |
| **2vik** | **A** | 1 | 122 | 122 | d.109.1.1 | 5.57 | 0.21 | 5.07 | 0.13 |
| **4ubp** | **A** | 1 | 100 | 100 | d.8.1.1 | 3.95 | 0.16 | 3.68 | 0.11 |
| **5cro** | **A** | 1 | 55 | 55 | a.35.1.2 | 1.23 | 0.09 | 1.05 | 0.06 |

The *ab-initio* benchmark set comprises 62 small globular proteins. For each target, coordinate root-mean square deviation (crmsd) of the top 0.1% model is reported based on extensive Rosetta computations. Columns 7,8: the reference (nnmake) fragments, columns 9,10: fragments selected by the new algorithm ; avg and sdev are mean and standard deviation from 50-fold 0.1 percentile bootstrap estimation (see also Figure 6).
